# The Cell Wall Protein Ecm33 of *Candida albicans* is Involved in Chronological Life Span, Morphogenesis, Cell Wall Regeneration, Stress Tolerance, and Host–Cell Interaction

**DOI:** 10.3389/fmicb.2016.00064

**Published:** 2016-02-02

**Authors:** Ana Gil-Bona, Jose A. Reales-Calderon, Claudia M. Parra-Giraldo, Raquel Martinez-Lopez, Lucia Monteoliva, Concha Gil

**Affiliations:** ^1^Departamento de Microbiología II, Facultad de Farmacia, Universidad Complutense de MadridMadrid, Spain; ^2^Instituto Ramón y Cajal de Investigación Sanitaria (IRYCIS)Madrid, Spain

**Keywords:** *Candida albicans*, Ecm33, stress response, cell wall regeneration, veil growth, chronological lifespan, host–cell interaction

## Abstract

Ecm33 is a glycosylphosphatidylinositol-anchored protein in the human pathogen *Candida albicans*. This protein is known to be involved in fungal cell wall integrity (CWI) and is also critical for normal virulence in the mouse model of hematogenously disseminated candidiasis, but its function remains unknown. In this work, several phenotypic analyses of the *C. albicans ecm33/ecm33* mutant (RML2U) were performed. We observed that RML2U displays the inability of protoplast to regenerate the cell wall, activation of the CWI pathway, hypersensitivity to temperature, osmotic and oxidative stresses and a shortened chronological lifespan. During the exponential and stationary culture phases, nuclear and actin staining revealed the possible arrest of the cell cycle in RML2U cells. Interestingly, a “veil growth,” never previously described in *C. albicans*, was serendipitously observed under static stationary cells. The cells that formed this structure were also observed in cornmeal liquid cultures. These cells are giant, round cells, without DNA, and contain large vacuoles, similar to autophagic cells observed in other fungi. Furthermore, RML2U was phagocytozed more than the wild-type strain by macrophages at earlier time points, but the damage caused to the mouse cells was less than with the wild-type strain. Additionally, the percentage of RML2U apoptotic cells after interaction with macrophages was fewer than in the wild-type strain.

## Introduction

The fungal cell wall is an essential organelle that is required for the maintainance of cell integrity and also plays an important role in primary interactions between pathogenic fungi and their hosts. Different analyses have demonstrated its importance as a protective barrier against a wide range of environmental conditions, such as temperature, oxidative and osmotic stresses. The cell wall is essential for the virulence of pathogenic fungi, since it provides adhesive properties and protection against host defense mechanisms. The composition of the *Candida albicans* cell wall consists of β-1,6-glucan, β-1,3-glucan and chitin, as well as different attached proteins, including glycosylphosphatidylinositol (GPI) proteins (Chaffin, 2008; Free, 2013). These GPI proteins contain a C-terminal domain that allows for linkage to a GPI anchor and might target proteins to the membrane or the cell wall.

The *C. albicans ECM33* gene encodes a GPI-anchored protein of this human pathogen. The Ecm33 protein was detected in the *C. albicans* cell wall, plasma membrane, extracellular vesicles, and soluble extracellular medium by proteomic analysis, revealing its high abundance (Castillo et al., 2008; Cabezón et al., 2009; Gil-Bona et al., 2015a) and it has also been detected by cell surface shaving analysis of yeast, hyphae, and biofilms (Vialas et al., 2012; Gil-Bona et al., 2015c). Although its function is unknown, it is highly important in cell wall morphology and virulence. The *ecm33△* null mutant (RMLU2) displays cell wall defects such as an enhanced sensitivity to cell wall-perturbing agents such as calcofluor white, Congo red and hygromycin B, an abnormal electron-dense outer mannoprotein layer and an aberrant surface localization of the adhesin Als1, together with defects during the yeast-to-hyphae transition (Martínez-Lopez et al., 2004, 2006). Recent proteomic analysis of the extracellular medium of RML2U relates Ecm33 to the proper functioning of the classical secretion pathway and to the composition, shape, and quantity of extracellular vesicles (Gil-Bona et al., 2015b). The secretory aspartyl proteinases, particularly Sap2, play important roles in vaginitis in mice (Pericolini et al., 2015). Sap2 secretion was compromised in the *ecm33△* mutant and negatively affects bovine serum albumin (BSA) degradation when BSA is used as the sole nitrogen source. Additionally, RML2U causes an irregular protein trafficking to the medium that might contribute to the avirulence of RML2U in a mouse model of systemic infection and to the reduced capacity to invade and damage endothelial cells and oral epithelial cells (Martínez-Lopez et al., 2004, 2006). RML2U cells are also sensitive to rapamycin, the inhibitor of the Target of Rapamycin (TOR) pathway, suggesting a relationship between the TOR pathway and Ecm33 (Gil-Bona et al., 2015b). The TOR kinase mediates important cellular responses that are implicated in extended longevity, metabolism and morphogenesis, including stress responses, autophagy and actin organization, among others (Wullschleger et al., 2006; Kaeberlein et al., 2007). Moreover, there is evidence of crosstalk between the TOR and cell wall integrity (CWI) pathways (Fuchs and Mylonakis, 2009).

The connection of Ecm33 in fungi with CWI is known (Martínez-Lopez et al., 2004; Pardo et al., 2004), but its contribution to stress tolerance is largely unknown in *C. albicans*. Recently a characterization study of two Ecm33 orthologs in *Beauveria bassiana* and *Metarhizium robertsii* was published, in which the relationship of this protein with multi-stress tolerance was demonstrated (Chen et al., 2014). However, in contrast with previous studies in *C. albicans*, these orthologs showed no relationship with virulence in a larvae assay. To clarify these results and to extend the study of Ecm33, this study characterizes *C. albicans* Ecm33 functions via a range of phenotypic analyses of the *ecm33Δ* mutant, RML2U, and its involvement in longevity and in the engulfment by macrophages.

## Materials and Methods

### Microorganisms and Culture Conditions

*Candida albicans* SC5314 (wild type; Gillum et al., 1984) was used to generate the RML2U mutant strain (*ecm33*△*::hisG/ecm33△::hisG ura3*△*::imm434/ura3*△- *::imm434::URA3*) and the doubled-complemented strain RML4U (*ecm33*△*::hisG::ECM33-cat/ecm33*△*::hisG::ECM33-cat ura3*△::*imm434-/ura3*△*::imm434::URA3*; Martínez-Lopez et al., 2006). *C. albicans* cell wall mutants used in the rapamycin assay were acquire from Noble collection (Noble et al., 2010) stored in the Fungal Genetics Stock Center (Kansas City, MO, USA; McCluskey et al., 2010). *C. albicans* strains were maintained on YPD (1% yeast extract, 2% peptone, and 2% glucose) agar plates at 30°C. For chronological life span (CLS) assays yeast cells were grown in synthetic defined (SD) cultures (20 g/L glucose, 5 g/L ammonium sulfate, 1.7 g/L nitrogen base, and 2.2 g/L amino acids mix) at 30°C. Cornmeal growth was in cornmeal media (2% commercial cornmeal) at 37°C 200 rpm shaking. For interaction and phagocytosis assays, RAW 264.7 murine macrophages were cultured in RPMI 1640 medium supplemented with antibiotics (penicillin 100 U/ml and streptomycin 100 μg/ml), L-Glutamine (2 mM) and 10% heat-inactivated fetal bovine serum (FBS) at 37°C in a humidified atmosphere containing 5% CO_2_.

### Cell Wall Regeneration

*Candida* protoplast yeast cells were prepared according to previous work (Pitarch et al., 2006). Cells were grown in YPD medium until OD_600_ 0.8–1.2, washed and incubated at 30°C 80 rpm in a pretreatment solution (10 mM Tris-HCl, pH 9.0, 5 mM EDTA, 1% v/v 2-mercaptoethanol) for 30 min. Then, 5 × 10^8^ cells/ml were resuspended in a solution containing 1 M sorbitol and 30 μg/ml glusulase (Du Pont^®^) and maintained with gentle shaking until protoplast were obtained. After five washes, protoplasts were induced to regenerate their cell walls in Lee medium (Lee et al., 1975) containing 1 M sorbitol at 30°C with gentle shaking (80 rpm) for 30 min and 3 and 24 h. The cell wall regeneration was observed by scanning electron microscopy (SEM) and osmotic fragility of regenerating protoplast were determined as reported previously (Nishiyama et al., 1995) with several modification. Briefly, the osmotic fragility of regenerating protoplast were determined at 0 min, 30 min, 3 h and 24 h by counting colony-forming units (CFUs) after resuspending a portion of the cells on phosphate buffer (PBS) with or without 1 M sorbitol for 3 min, and the resulting suspension was diluted and plated in triplicate on YPD agar containing 1 M sorbitol. After 72 h incubation at 30°C, visible colonies were counted. The percentage of CFUs was calculated using like 100% the number of CFUs counted from the solution with sorbitol. Three biological replicates were carried out and the statistical significance of the differences in CFU numbers was evaluated by using the Student *t*-test.

### Protein Extracts and Immunoblot Analysis

Yeast strains were grown overnight at 37°C in 20 ml YPD cultures. Cells extracts were obtained by suspending cells in cold lysis buffer [50 mM Tris/HCl, pH 7.5, 10% glycerol (v/v), 1% Triton X-100, 0.1% SDS, 150 mM sodium chloride, 50 mM sodium fluoride, 1 mM sodium orthovanadate, 50 mM β-glycerol phosphate, 5 mM sodium pyrophosphate, 5 mM EDTA, pH 8, 1 mM PMSF, 25 μg ml^-1^ *N*-tosyl-L-lysine chloromethyl ketone hydrochloride (TLCK), 25 μg ml^-1^ *N*-*p*-tosyl-L-phenylalanine chloromethyl ketone (TPCK), 25 μg ml^-1^ pepstatin A, 25 μg ml^-1^ leupeptin, 25 μg ml^-1^ antipain, and 25 μg ml^-1^ aprotinin] and lysed mechanically using glass beads in a fast-prep cell breaker applying three 35 s rounds at 5.5 speed with intermediate ice coolings. Cells extracts were clarified by centrifugation, and the supernatants were collected and stored at -80°C. The protein concentration was measured by Bradford protein assay. Equal amounts of proteins (80 μg per lane), were loaded onto 10% SDS-polyacrylamide gel, separated and transferred to nitrocellulose membranes and blocked with 5% milk. Reversible Ponceau red staining was applied to check protein transferring to the membranes. Western blots were probed with anti-phospho-p44/42 MAP kinase (Thr202/Tyr204; Cell Signaling Technology, Inc.) that recognizes the phosphorylated form of Mkc1 and Cek1 kinases, and anti-Hog1 (Santa Cruz Biotechnology) using 1:2000 dilutions. Primary antibodies were detected using fluorescently labeled secondary antibody IRDye 800 goat antirabbit IgG (LI-COR Biosciences) at 1:5000. The Western blotting was performed with the Odyssey system (LI-COR Biosciences, Nebraska).

### Stress-Related Phenotypic Assays

Drop tests were performed by spotting serial dilutions of cells onto agar plates and YPD agar plates supplemented with sorbitol (1.5 M), KCl (1 M), NaCl (1 M), caffeine, diamide (2 mM), menadione (0.2 mM), and rapamycin (0.1 μg/ml). Hydrogen peroxide stress was tested adding H_2_O_2_ 100 mM to *C. albicans* growing cells in YPD medium at an OD_600_ 0.8 at 30°C and spotted onto YPD plates at different times: 2, 5, 10, 15, 30, 60, and 90 min. Plates were incubated 24–48 h at 30°C. For heat-shock stress YPD plates were incubated at different temperatures (30, 37, 42, and 45°C).

### Growth Curve and Growth Rate Determination

Overnight YPD cultures were used to inoculate 100 ml YPD at an OD_600_ of 0.1. Cultures were grown at 30°C with shaking. OD measurements were taken every hour to generate growth curves. Doubling times of each strain were calculated using time points within the logarithmic phase of growth. This assay was repeated three times.

### Staining and Fluorescent Image Analysis

For viability assay, Propidium Iodide was added to cultures to a final concentration 10 μg/ml and incubated for 10 min at room temperature. Cells were washed and resuspended in PBS. For chitin staining, calcofluor white was added directly to the culture medium to a final concentration 5 μg/ml. After 10 min cells were washed with PBS and observed by fluorescent microscopy. For DNA-specific fluorescent probe, DAPI (4′,6-diamidino-2-phenylindole) was added to the cell culture at a final concentration 0.5 μg/ml, incubated 5 min in the dark and then rinsed with PBS and observed by fluorescent microscopy. To detect actin, cells were stained as described previously (Hausauer et al., 2005). Briefly, *C. albicans* cells were fixed in 3.7% formaldehyde for 30 min, followed by incubation in PK buffer (50 mM potassium phosphate, pH 3.6) containing 3.7% formaldehyde for an additional 60 min. Formaldehyde-fixed cells were harvested by centrifugation, incubated in PK buffer containing 0.1% Triton X-100 for 30 min, washed twice in PBS, and incubated overnight in PBS containing 2 U of Alexa Fluor 568 phalloidin (Molecular Probes, Eugene, OR, USA) at 4°C. Cells were harvested by centrifugation and resuspended in PBS prior to fluorescence microscopy. For vacuolar membrane staining, 0.2 μl FM4-64 dye (Invitrogen T3166 1 mg) were added and incubated o/n at 37°C shaking. Cells were washed and resuspended in PBS.

### Chronological Life Span

Yeast CLS was measured as previously described (Wei et al., 2008). Overnight liquid SD cultures were diluted 1:200 in 10 ml fresh SD medium and were maintained at 30°C with shaking. This time point was considered day 0. Every 3 days, aliquots from the culture were properly diluted and plated on to YPD plates. The YPD plates were incubated at 30°C for 3 days and viability was checked by CFUs. Viability at day 3, when the yeast had reached the stationary phase, was considered to be the initial survival (100%). For extreme calorie restriction (CR)/starvation, cells from 3 days-old SD culture were washed three times with water, and resuspended in 10 ml water. This time point was considered day 0. Water cultures were maintained at 30°C with shaking. Every 3 days, cells from the water cultures were washed to remove nutrients released from dead cells and plated onto YPD plates. For CR modeled by glucose reduction, overnight SD culture was diluted (1:200) into fresh 0.5% glucose SD medium. The protocol followed is the same as for yeast CLS. All dilutions were plated three times and several dilutions were made. The complete experiment was repeated three times. The values represented correspond to the average of the three replicates.

### Induced Apoptosis by Interaction with Macrophages

Macrophages were grown in 6-well plates at a cell density of 6 × 10^6^ cells/well. *C. albicans* cells were added at a ratio 1:1 and co-incubated at 37°C with 5% CO_2_. Different apoptotic markers were analyzed as previously described Cabezón et al. (under revision). Each experiment was repeated three times for each interaction time point and the statistical significance relative to the RML2U control cells without macrophages were performed by Student *t*-test. Briefly, intracellular reactive oxygen species (ROS) were detected in control *Candida* cells and cells co-incubated with macrophages (3, 6, and 8 h) by adding 5 μg/ml dihydrorhodamine (DHR) 123 (Sigma-Aldrich) 30 min before the end of each experiment. *In vivo* measurement of caspase-like enzymatic activity was performed washing the cells in PBS and resuspended in 10 μM/ml of staining solution containing fluorescein isothiocyanate FITC-VAD-FMK (CaspACET, Promega) for 20 min at 37°C after each interaction time point (3, 6, and 8 h). After each interaction time point, cells were washed with water in order to lysate the macrophages and cells were recovered and evaluated using a fluorescence microscopy.

### *C. albicans* Phagocytosis Assay

For the phagocytosis assay, macrophages were plated onto 18-mm glass sterile coverslips placed in 24-well plates. *C. albicans* strains were pre-labeled with 1 μM Oregon Green 488 (Molecular Probes) in the dark with gentle shaking at 30°C for 1 h. Macrophages were confronted with the yeast at a ratio 1:1 at 37°C and 5% CO_2_. Interaction was stopped after 45 min, 1.5 and 3 h and cells were then washed with ice-cold PBS and fixed in 4% paraformaldehyde for 30 min. To distinguish between internalized and attached/non-ingested yeasts, *C. albicans* cells were counterstained with 2.5 M calcofluor white (Sigma) for 15 min in the dark. The number of ingested cells (green fluorescence) and/or adhered/non-ingested (calcofluor white blue fluorescence) were quantified by fluorescence microscopy with FITC and UV (Fernández-Arenas et al., 2007). Three different replicates with two different slides were prepared for each time point. At least 400 *C. albicans* cells were counted per slide, and results were expressed as the percentage of yeasts internalized by macrophages. Statistical analyses were performed using the Student *t*-test.

### Cytotoxicity Measurement

RAW 264.7 cells were seeded onto 24-well plastic plates at a density of 1 × 10^6^ cells/well in RPMI 1640 complete medium and incubated 24 h at 37°C in a humidified atmosphere containing 5% CO_2_. Then, macrophages were co-incubated [in a new complete media without phenol red (pH indicator) to avoid the background in the lactate dehydrogenase (LDH) test] with *C. albicans* yeast strains at a ratio 1:1 during 3 and 8 h. Staurosporine 5 mM was used as a positive control. After the incubation, LDH was measured with the Cytotoxicity Detection Kit^PLUS^ (Roche) according to the manufacturer’s protocol. Statistical analyses were performed using the Student *t*-test.

### Microscopy Techniques

For fluorescence microscopy, a fluorescence microscope NIKON ECLIPSE TE2000-U, connected to a high resolution HAMAMATSU ORCA-ER camera was used. For SEM, used to visualize protoplast cell wall regeneration, cells were fixed with 2% (v/v) glutaraldehyde in 0.1 M cacodylate buffer containing 1 M sorbitol at 4°C, overnight. Post-fixation was carried out for 2 h at room temperature with 2% osmium tetroxide in 0.1 M cacodylate buffer (pH 7.2). Initial dehydration was accomplished in a graded ethanol series. Then, samples were dehydrated with acetone until dried by the critical point method in liquid CO_2_ (Balzers^®^ CPD 030). Subsequently, the specimens of the different strains were coated with graphite and gold in a vacuum evaporator (EMITECH SCD 004) and examined with a SEM JEOL Observations were carried out in JEOL JSM-6400 microscope SEM. For transmission electron microscopy (TEM), used to observe cell wall, cells were fixed in 4% paraformaldehyde, 1%, glutaraldehyde, and 0.1% PBS overnight at 4°C. Samples were incubated for 90 min in 2% osmium tetroxide and then serially dehydrated in ethanol and embebbed in EMBed-812 resin (Electron Microscopy Sciences). Thin sections (50–70 nm) were obtained by ultracut and observed in JEOL JEM 1010 TEM.

## Results

### RML2U has the Cell Integrity Pathway Activated and its Protoplast are Unabled to Regenerate the Cell Wall

**FIGURE 1:**
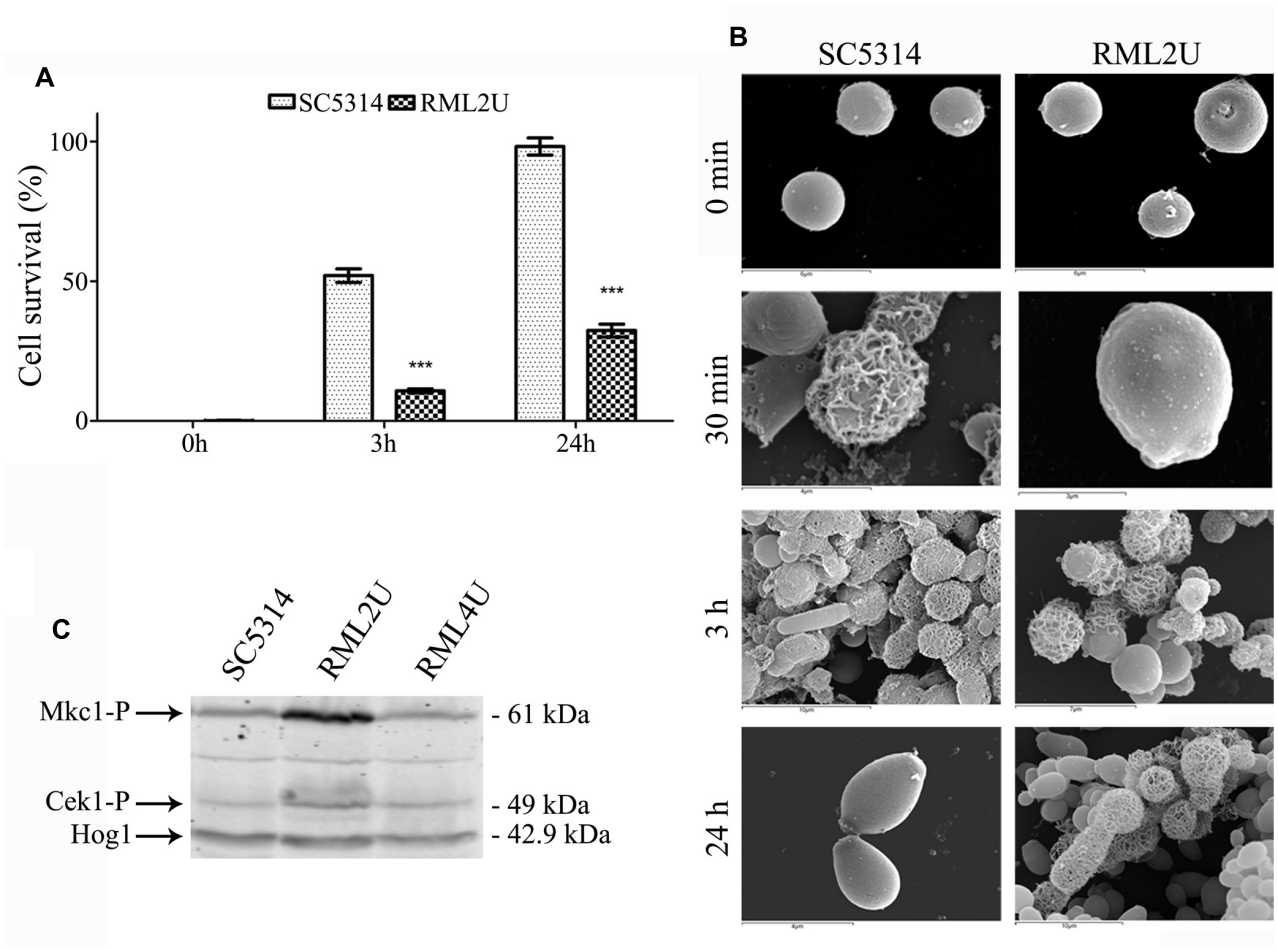
**Protoplast regeneration and CWI activation. (A)** Comparison of the percentage of viable counts of osmotically stabilized shocked cultures of *Candida albicans* cells. Statistically significant differences relative to the wild-type are indicated (^∗∗∗^*p* < 0.001). **(B)** Scanning electron microscopy (SEM) of freshly prepared protoplast after 0 min, 30 min, 3 h and 24 h of cell wall regeneration in Lee medium supplemented with sorbitol. **(C)** Mkc1 is activated in the RML2U strain. Western blotting analysis of protein extracts from the indicated strains.

The ability of RML2U to regenerate the protoplast cell wall was examined to study Ecm33 involvement in the synthesis and organization of the cell wall (Martínez-Lopez et al., 2004). Cells were treated with glusulase to obtain more than 90% protoplasts and these were incubated in regeneration conditions for 0, 3, or 24 h. The osmotic fragility of the regenerating protoplast cell wall was determined by colony counts on agar plates in the presence of stabilizer (1 M sorbitol) after resuspending the cells in PBS in the presence or absence of 1 M sorbitol. More than half of the SC5314 cells (52%) were osmotically stable after 3 h of regeneration, whereas only 10% of RML2U cells started to proliferate (**Figure 1A**). At the end of the study, after 24 h of regeneration, 98% of the SC5314 cells showed osmotic stability, compared with 32% of RML2U cells.

Protoplast regeneration was examined by SEM. Fresh protoplasts appeared as round cells with a smooth surface (**Figure 1B**). After 30 min of regeneration, a net of thick fibrils were irregularly distributed over the surface of most SC5314 protoplasts. After 3 h, most cells showed filamentous material and some cells showed complete protoplast regeneration. After 24 h of regeneration, all cells exhibited complete regeneration of the cell wall; however, RML2U was unable to regenerate the cell wall. A network of filamentous materials started to cover several protoplasts after 3 and 24 h of regeneration, some cells possessed complete cell wall but regeneration was incomplete for other protoplasts. The SEM images showed that some cells probably began to generate the cell wall, but were unable to complete the process (**Figure 1B**). Protoplast regeneration of RML2U appeared to be greatly delayed compared with that of the SC5314 strain and most cells were unable to complete the process, according to osmotic fragility data (**Figure 1**).

To understand whether the absence of *ECM33* is sensed by the MAP K pathway, the phosphorylation of Mkc1, the MAP kinase of the cell integrity pathway, and Cek1, the protein kinase part of a MAPK regulatory cascade, were analyzed by examining growing cells at 37°C in YPD. Commercial antibodies raised against the phosphorylated forms of p44-42 MAP kinases recognize the simultaneous phosphorylation of the threonine and tyrosine residues of the TEY signature of the Mkc1 and Cek1 proteins in *C. albicans* (Navarro-Garcia et al., 2005). Differences were clearly observed concerning the intensity of phosphorylation (**Figure 1C**). The intensity of the signal of the upper band (corresponded to Mkc1 61 kDa) was greater compared to that of the wild type strain. Notably, the signal decreased in the reintegrated strain, RML4U. The lower band corresponds to the phosphorylated form of Cek1 (49 kDa), and the signal was slightly increased compared to that in wild type and RML4U strains. Therefore, Mkc1 is phosphorylated in the absence of *ECM33*.

### RML2U is Hypersensitive to Temperature, Osmotic and Oxidative Stresses

The activation of cellular protection mechanisms represents an important survival strategy in yeast (Longo and Fabrizio, 2002). Furthermore, the CWI pathway crosstalks with other stress-response pathways, such as the TOR pathway (Fuchs and Mylonakis, 2009). To examine the potential role of Ecm33 in cellular protection and its relationship with other pathways, the cellular responses of RML2U to different types of stresses were compared with those of SC5314.

**FIGURE 2:**
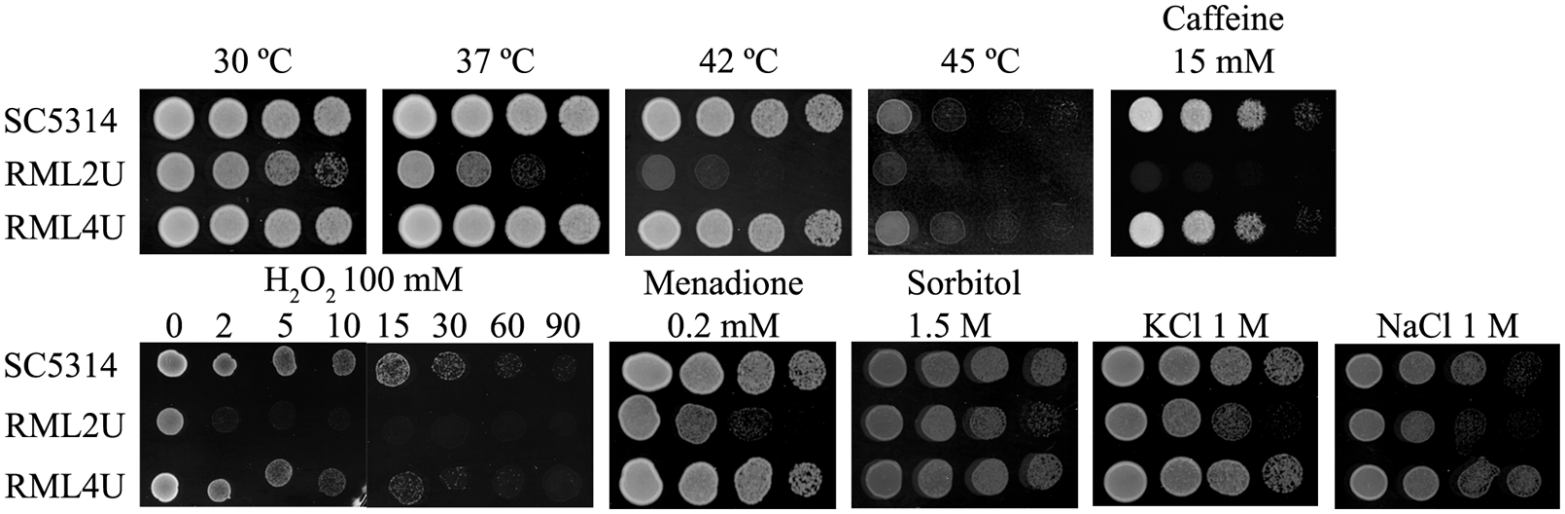
**Stress response.** Strains were grown in the presence of 100 mM hydrogen peroxide (H_2_O_2_), 0.2 mM menadione, 1.5 M sorbitol, 1 M KCl, 1 M NaCl and 15 mM Caffeine, and were heat shocked at 37, 42, and 45°C. Control plate was also performed at 30°C in YPD.

Deletion of *ECM33* affects the growth of *C. albicans* under oxidative, temperature, and osmotic stress. Oxidative stress induced by different exposure times to 100 mM hydrogen peroxide, resulted in a slight sensitivity in the RML2U strain and importantly, this phenotype was reversed upon reintroduction of the wild type *ECM33* gene into the *ecm33Δ/ecm33Δ* strain (RML4U; **Figure 2**), which was performed by growing *C. albicans* in the exponential phase (OD_600_ 0.8). Time 0 represents the culture before addition of the stress factor. The oxidative stress response was also tested by growing the strains on YPD agar supplemented with 0.2 mM menadione, another oxidative stress-inducing agent, which can generate ROS by redox cycling which leads to stress (**Figure 2**). No difference was observed in response to diamide, a thiol oxidant (data not shown).

Growth at high temperatures, from 37 to 45°C, seriously affected the growth of RML2U compared with SC5314 (**Figure 2**), which suggests that the mutant begins to display compromised growth at human body temperature. Notably, the deletion of *ECM33* resulted in impaired resistance to the osmotic stress-inducing agents’ sorbitol (1.5 M), NaCl (1 M), and KCl (1 M; **Figure 2**). The sensitivity to caffeine, a compound related to the CWI pathway, as well as to TOR function, was also tested. The RML2U strain showed an increase in the sensitivity to caffeine compared with SC5314 (**Figure 2**).

### Ecm33 is Necessary for Normal Morphology

The observation of several cultures of RML2U in standard growth conditions (YPD 30°C, 200 rpm shaking), showed a variety of different morphologies ranging from relatively normal to aberrant cells (Martínez-Lopez et al., 2004). Therefore, the morphology of this mutant was studied under several conditions using SC5314 as a control strain. Firstly, growth rates were determined to corroborate the data of Martínez-Lopez et al. (2006). The RML2U growth rate was approximately 1.5 times slower than that of SC5314.

**FIGURE 3:**
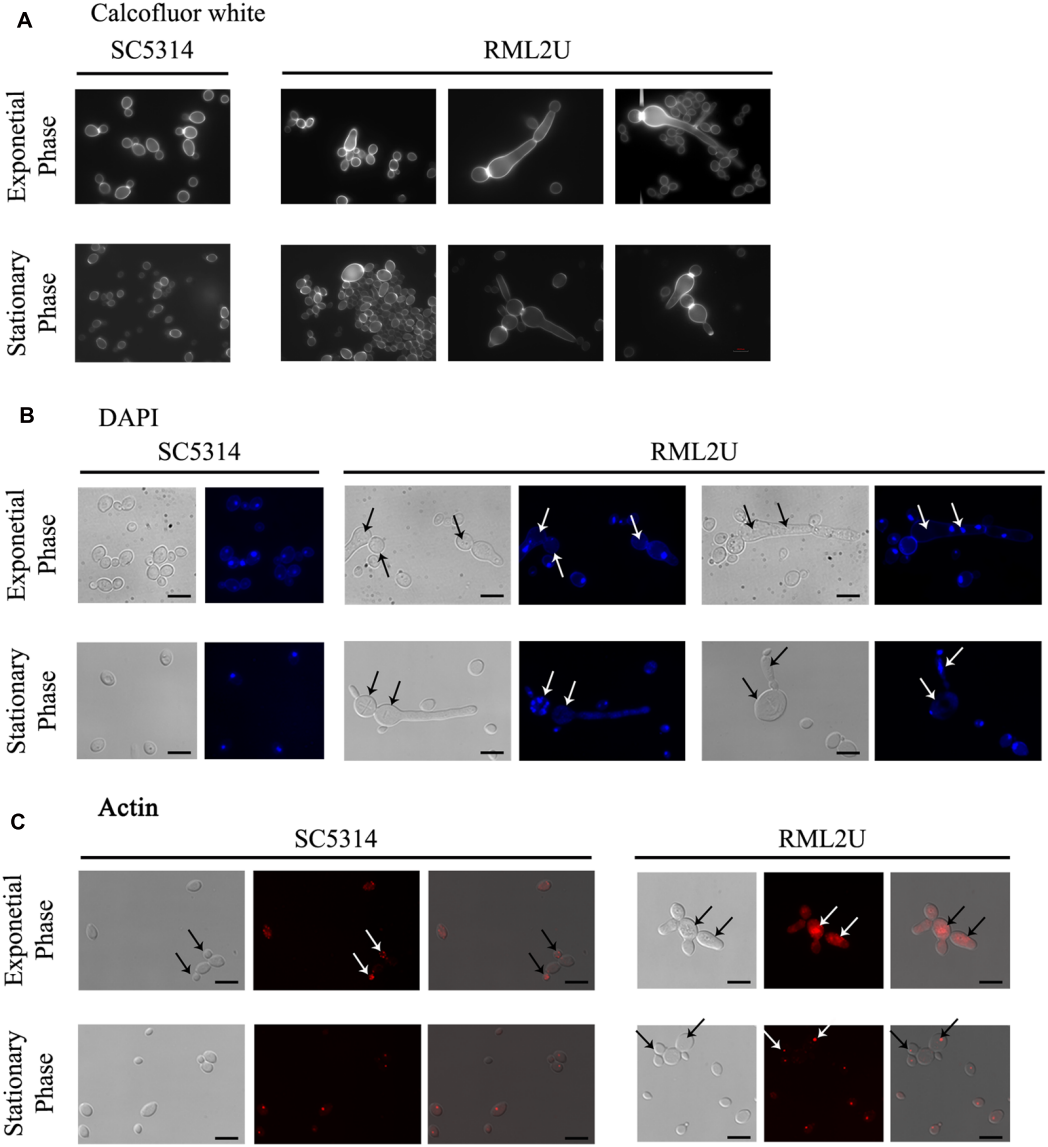
**Ecm33 is important for nuclear DNA division, septa distribution and actin polarization during *C. albicans* growth. (A)** calcofluor staining was used to visualize the chitin localized in the septum. **(B)** The nuclear DNA was observed by labeling with DAPI, a highly specific stain for DNA. Arrows indicate defects in the number of nuclei. **(C)** *C. albicans* strains were fixed and stained with Alexa phalloidin to visualize actin. Arrows in SC5314 indicate normal actin polarization in buds, compared to RML2U.

Previous study showed that RML2U cells had an aberrant morphology, which varied depending on the growth conditions and the culture medium. Staining with calcofluor white, a compound that binds to chitin or glucan polymer of the cell wall, showed uniformly stained material in wild type and large aggregates in RML2U (Martínez-Lopez et al., 2004). Different morphologies during growth in YPD medium at 30°C resulted from compromised *ECM33* function (**Figure 3** and Supplementary Figure S1A). The cell morphology of the exponential and stationary growth phases was analyzed to examine the response and phenotype of RML2U under these conditions in depth. For this purpose, calcofluor white was used to examine the distribution of septa by fluorescence microscopy. Some pseudohyphal-like RML2U cells were observed in the exponential phase (**Figure 3A**). Regular cells and large cells that were not separated from the mother cell were observed and the amount of chitin appeared to be higher in RML2U cells. These data were confirmed by flow cytometry and were twice as high as the values detected in SC5314 cells (data not shown). In addition, cells with aberrant morphologies, including non-separated cells and large cells, were counted and 10% showed some type of aberrant morphology (Supplementary Table S1). On the other hand, the localization of DNA and distribution of actin in the cells in each stage of growth were observed by specific dyes (**Figures 3B,C**). In the exponential phase, fewer than 1% of the total cells counted contained more than two nuclei between the septa and no DNA was detected in 2% of the observed cells (**Figure 3B** and Supplementary Table S1). Nuclear division was uncoupled in RML2U cells. Phalloidin staining revealed that actin was mislocalized in RML2U, and appeared large actin patches in the center and surrounding cells (**Figure 3C**). These results reveal a defect in the cell cycle. Furthermore, the altered morphology and cell wall organization of RML2U was analyzed in more detail by TEM. Sections of SC5314 cells showed a smooth cell wall layer with a thickness between 126 and 88 nm (Supplementary Figure S1C), but the RML2U cell wall was thicker than in SC5314, at 334.21–79.63 nm, corroborating the cytometry data.

**FIGURE 4:**
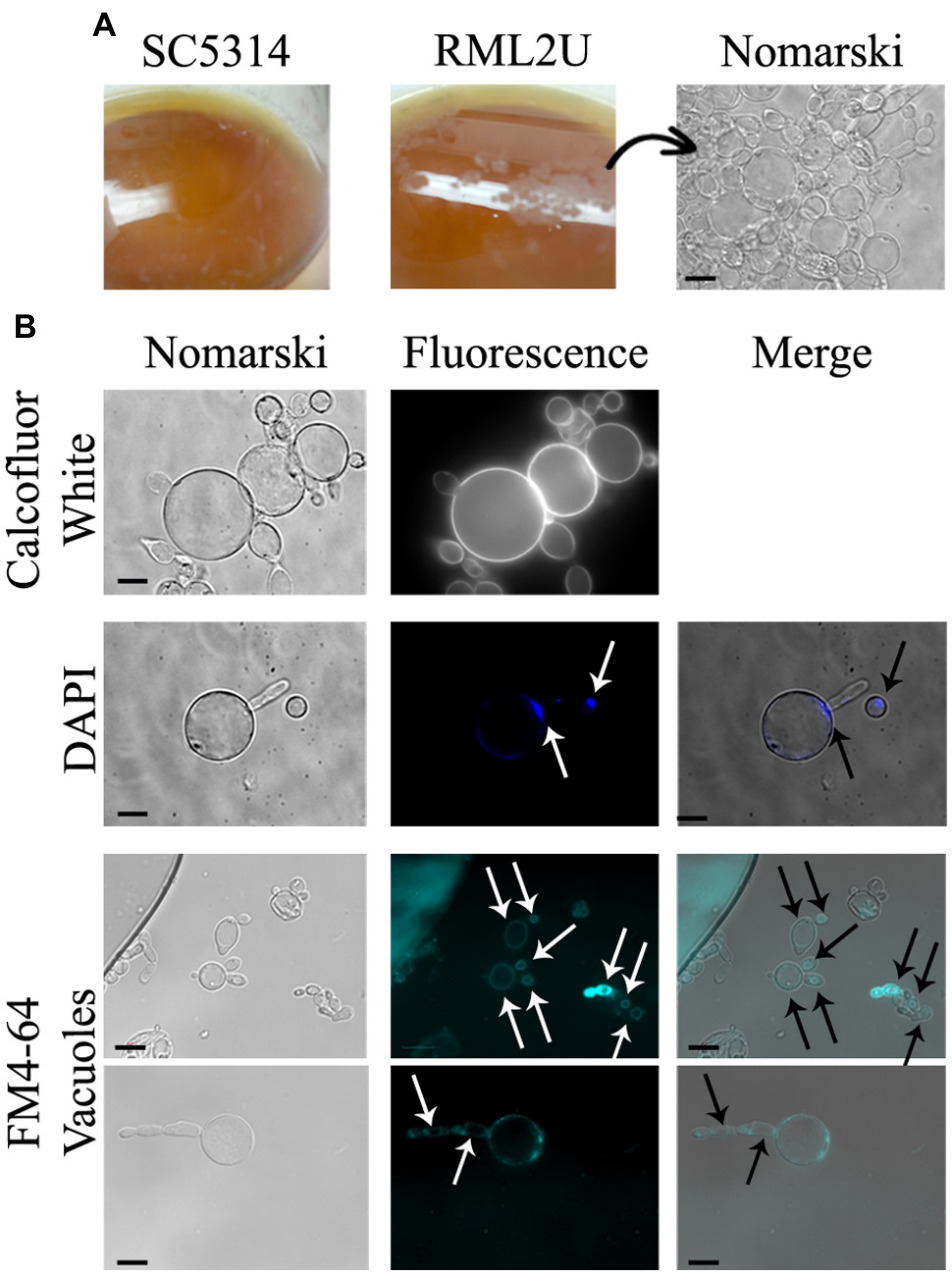
**Novel *C. albicans* “veil growth.” (A)** “Veil growth” was observed in stationary and static RML2U cultures and not in those of SC5314. **(B)** Different cell stainings were performed to clarify chitin distribution (calcofluor white staining), nuclear DNA composition (DAPI labeling) and to locate each cell vacuole (FM4-64).

The proportion of aberrant cells increased during the stationary phase and the different morphologies observed in these cultures are shown in **Figure 3**, including elongated cells and aggregates. calcofluor staining also showed unseparated cells and approximately 5% of non-nuclear cells were stained with DAPI (**Figures 3A,B**, and Supplementary Table S1). Moreover, a “veil growth,” never previously described for *C. albicans*, was serendipitously observed in RML2U stationary and static cultures and not in those of SC5314 at room temperature after weeks (**Figure 4A**). The microscopic analysis of the cells within this structure revealed giant cells with a round morphology and DAPI stain showed only slight fluorescence on the side of the cell (**Figures 4A,B**). calcofluor staining showed a homogenous distribution of chitin around the cells, which increased in amount, which also occurs in other RML2U conditions (**Figure 4B**). In addition, FM 4–64 staining was performed, which is a vital stain for the vacuolar membrane in yeast (Vida and Emr, 1995). A large cellular compartment was observed in cells that recovered from the “veil,” which completely occupied the cell (**Figure 4B**). In several cells, an irregular allocation of this marker was observed, suggesting aberrant internal structures or no living cells.

**FIGURE 5:**
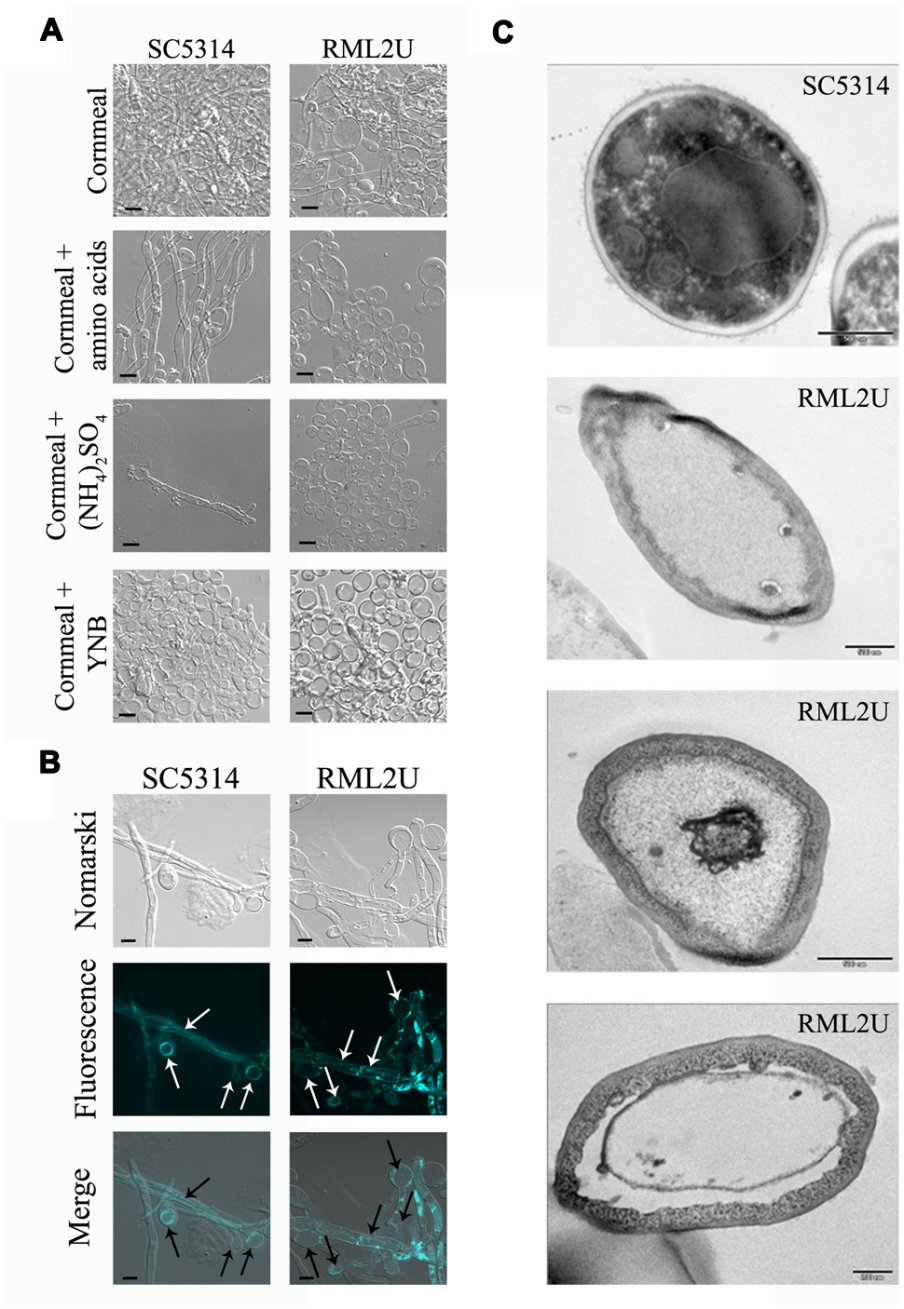
**Cornmeal liquid medium mimics the “veil growth” cell phenotype. (A)** Amino acids, ammonium (NH_4_)_2_SO_4_, and yeast nitrogen base (YNB) were added to the cornmeal medium to observe their influence on the RML2U cell phenotype. **(B)** The cell vacuole was observed by FM4-64 labeling. **(C)** TEM images of SC5314 and RML2U cells from cornmeal culture. Scale bar: 500 and 200 nm (the last image of RML2U).

Similar cells with this shape were observed on cornmeal agar (data not shown), a medium used to study chlamydospore production in *C. albicans* (Pollack and Benham, 1957). Cells that grew in liquid 10% cornmeal were large and, as in “veil growth,” contained large vacuoles (**Figures 5A,B**) and no DAPI or propidium iodide-positive cells were observed (data not shown). SC5314 cells displayed hyphal growth in liquid cornmeal medium. Supplementing the medium with amino acids, ammonium sulfate, or yeast nitrogen base did not result in cells with a normal phenotype, moreover, is toward more visible even in the wild type. Cells that grew in this medium were observed by TEM and showed abnormal structures, with a large cell wall, massive vacuolization, translucent cytoplasm, and heterogeneous sizes compared with SC5314 cells (**Figure 5C**).

### RML2U Cells show a Shorter Chronological Life Span

**FIGURE 6:**
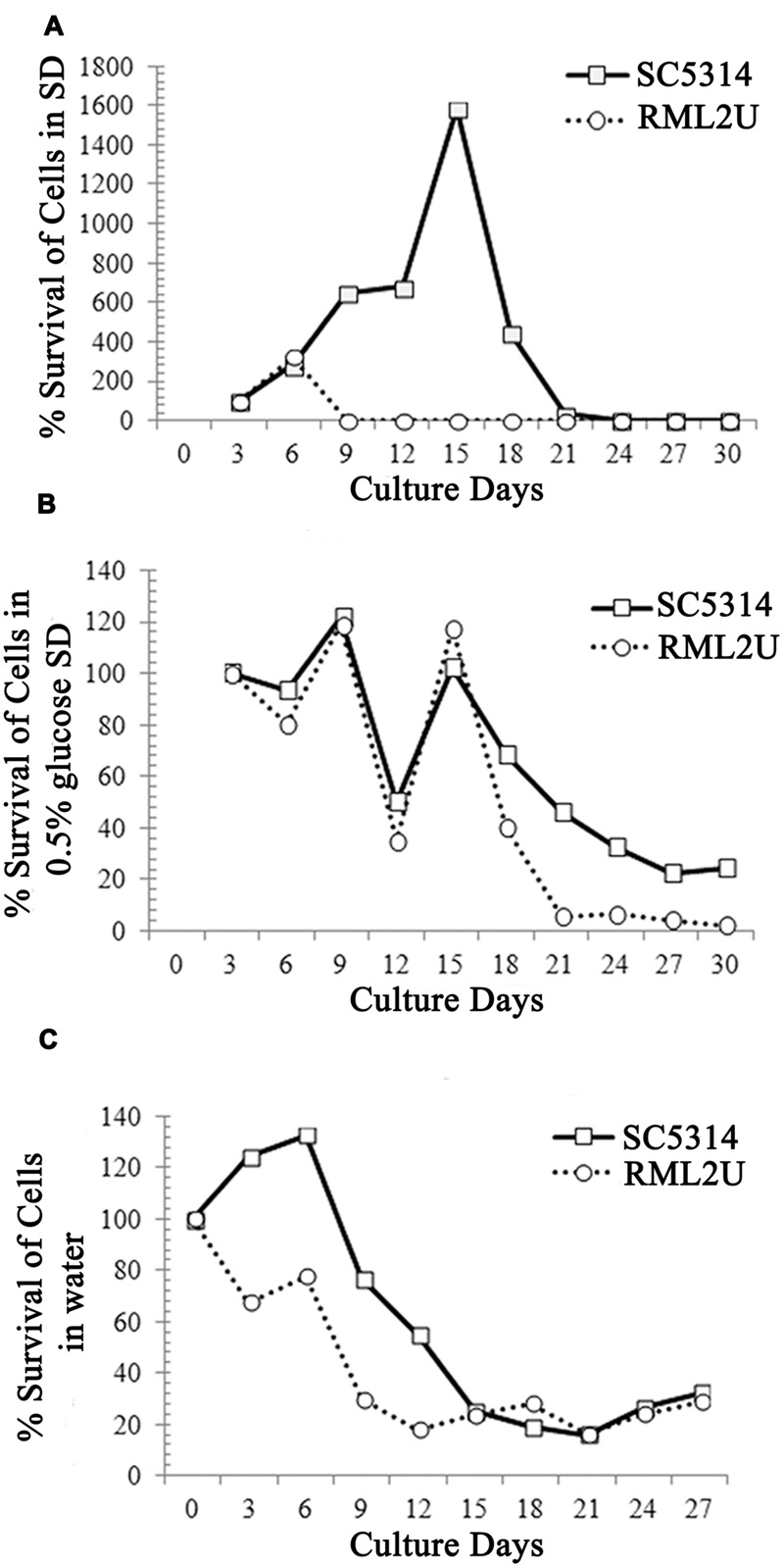
**Ecm33 possitively influences the chronological life span of *C. albicans*. (A)** SC5314 and RML2U were logarithmically growth in SD medium at 30°C. Samples were taken at the indicated times to determine CLS, as described in Section “Materials and Methods.” **(B)** CLS under calorie restriction by glucose reduction (0.5% glucose SD). **(C)** Extreme calorie restriction in which cultures were transferred to water on day 3 further extends the life span of RML2U.

Previous studies showed that defects in the TOR signaling pathway can extend the CLS of *Saccharomyces cerevisiae* (Wei et al., 2008). Recently, it has been shown that Ecm33 is related to the TOR pathway because of its sensitivity to rapamycin (Gil-Bona et al., 2015b). To determine whether the CLS is affected in the RML2U strain, growth in SD medium plus amino acids and glucose was compared with that of SC5314. The CLS measures the length of time that cells remain viable in the stationary phase. The number of colonies on day 3 was considered to represent the initial survival rate (100%). Cells of each strain were collected every 3 days and were plated on YPD agar for 48 h to determine the percentage survival. The CLS was different in RML2U cells than in SC5314 cells. Both strains contained an almost similar percentage of surviving cells by day 30, but the behavior of both strains differed considerably during the period of study (**Figure 6A** and Supplementary Table S2). On day 9, the percentage survival of RML2U cells decreased to 4%, whereas that of SC5314 increased to day 18 and then decreased more gradually. These data suggest that Ecm33 is necessary for cell survival.

In addition, extreme CR/starvation, in which stationary phase cells were transferred to water, doubled the mean life span of wild type yeast (Fabrizio et al., 2004, 2005). To understand the role of Ecm33 in CR, we monitored RML2U survival in CLS under CR modeled by glucose reduction (0.5% glucose SD medium) and in water. The behavior of the strains under glucose reduction CR (0.5% glucose) was different from that under CLS (**Figure 6B**). At the end of the study, on day 30, 10-fold fewer RML2U cells survived (2.4%) than SC5314 cells (24%). The decrease and increase in the survival rate of both strains was gradual, but the percentage of cells was lower in RML2U from day 18 (40%) to 30 (2.4%) compared to SC5314 (68 and 24%). Unexpectedly, the percentage of surviving cells of SC5314 and RML2U by day 30 in starvation/extreme CR conditions was almost similar, at about 30% (**Figure 6C**). At day 3, the percentage survival of RML2U decreased to 70%, then increased to 77% at day 6 and decrease more gradually from day 9 (29%) to day 30 (24%). The decrease in the percentage survival of SC5314 cells was more gradual from days 9 to 30. Taken together, these data suggest that Ecm33 is required for longevity, whereas it is dispensable for survival in conditions of extreme CR.

### Macrophage-*C. albicans* Interaction: Phagocytosis, Cytotoxicity, and Apoptosis

**FIGURE 7:**
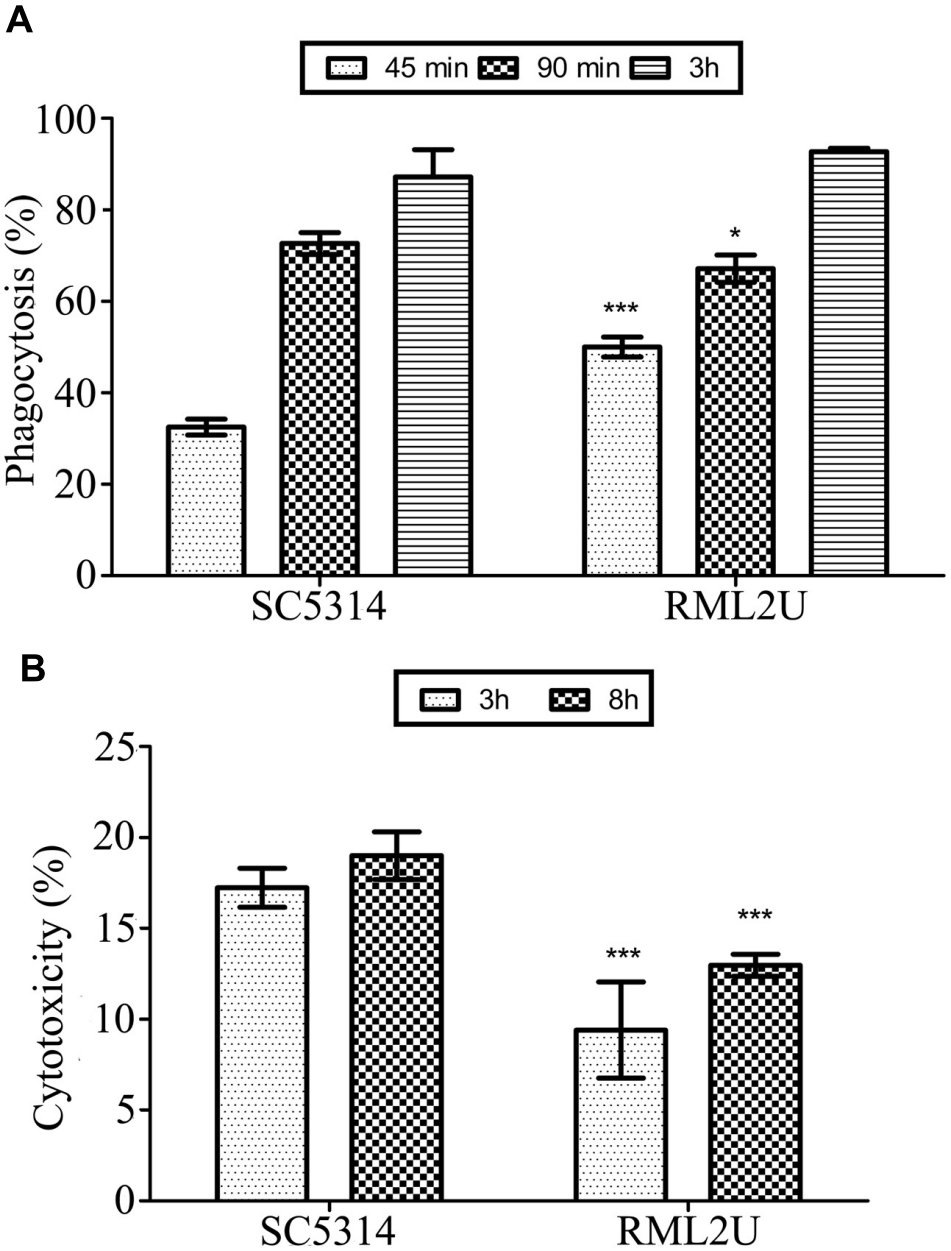
**Murine macrophage response to *C. albicans* strains. (A)** Quantification of phagocytosis of *C. albicans* yeast at 45 min, 90 min and 3 h following co-incubation. **(B)** Cytotoxicity of the SC5314 and RML2U strains in RAW 264.7 macrophages at 3 and 8 h following co-incubation. Data are represented as the mean ± SD (*n* = 3), and statistically significant differences relative to the wild-type are indicated (^∗^*p* < 0.05; ^∗∗∗^*p* < 0.001).

To understand the effect of *C. albicans ECM33* on the immunological response, some effector functions were assessed in mouse macrophages. Phagocytosis by RAW 264.7 macrophages were evaluated at a ratio of 1:1 and at different interaction times. Phagocytosis was measured at 45 min, 90 min and 3 h after the start of the interaction. **Figure 7A** shows that the recognition and engulfment of RML2U at 45 min was statistically significantly higher than for SC5314 cells and no difference was observed at later time points.

To determine the ability of the SC5314 and RML2U *C. albicans* strains to damage RAW 264.7 macrophages, the LDH cytotoxicity detection kit was used to measure the amount of LDH released into the medium from damaged cells. **Figure 7B** shows that RML2U consistently caused significantly less damage to the macrophages than SC5314 and was more than 30% less cytotoxic, at the different time points studied.

**FIGURE 8:**
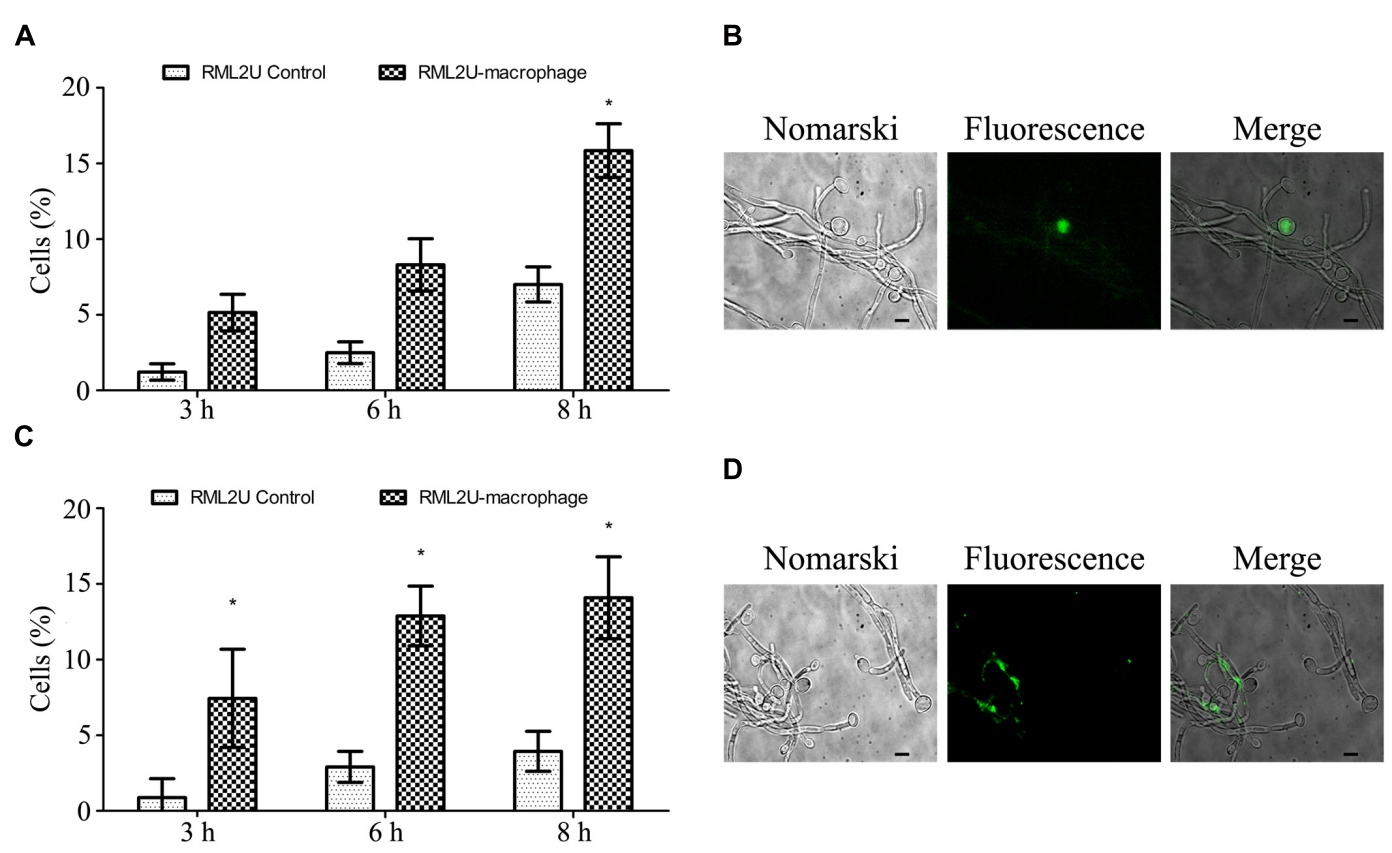
**Measurement of the apoptotic status of *C. albicans* strains after the interaction with murine macrophages. (A)** Percentage of *C. albicans* cells that contain ROS after 3, 6, and 8 h of macrophage interaction. **(B)** Representative fluorescence images of *C. albicans* culture stained with DHR 123. **(C)** Percentage of *C. albicans* cells with active caspase revealed by the caspase inhibitor VAD-FMK-FITC. **(D)** Representative fluorescence micrographs showing caspase activity in *C. albicans* cells. Statistically significant differences relative to the RML2U control are indicated (^∗^*p* < 0.05).

We propose a model of *C. albicans*-macrophage interaction in which more than 30% of SC5314 *C. albicans* cells ingested by RAW 264.7 died through apoptosis after 12 h of interaction (Cabezón et al., under revision). Therefore, the apoptotic status of RML2U at different time points of interaction with macrophages was analyzed. Two apoptotic markers were analyzed: the accumulation of ROS and caspase-like enzymatic activity. As shown in **Figures 8A,B**, significantly more cells in the RML2U interaction samples (16%) accumulated ROS than the control cells (7%) or *C. albicans* RML2U control cells without macrophages. The percentage of RML2U cells containing activated caspase increased after 3 h of interaction and this percentage (>15%) was maintained throughout the time-period studied (**Figures 8C,D**).

## Discussion

Fungal cell walls are vital organelles that determine cell shape, protect against physical injury and other stresses imposed by the external environment, and are essential for host–pathogen interactions (Klis et al., 2009). The integrity of the cell wall is very important for the survival of the microorganism and stress damage actives mechanisms developed by the cell to repair and reinforce the wall through cell wall biosynthesis and the integration of cell wall components into the wall, via activation of the CWI pathway (Lagorce et al., 2003). The activation of this pathway is not restricted to an individual stimulus and can be elicited by a number of events. Crosstalk between the CWI pathway and other stress-response pathways enable the CWI pathway to respond to diverse stress events with responses that appropriately alleviate cellular stress (Fuchs and Mylonakis, 2009). Here, the activation of the CWI pathway was studied by the phosphorylation of the 42–44 MAK kinases. The results agree with those of previous studies, which showed that Ecm33 is required for CWI and morphogenesis in *S. cerevisiae* and *C. albicans* (Martínez-Lopez et al., 2004; Pardo et al., 2004; Gil-Bona et al., 2015b). However, there is evidence for crosstalk between TOR-mediating signaling and the CWI pathway in *S. cerevisiae* (Torres et al., 2002). Previous studies showed the sensitivity of RML2U to rapamycin, the inhibitor of TOR kinase, which connected Ecm33 with this pathway (Gil-Bona et al., 2015b). In addition, to rule out that this sensitivity was due to cell wall damage, the sensitivity to rapamycin was tested in other *C. albicans* cell wall protein mutants, such as *mkc1/mkc1* and *cht2/cht2*. These mutants showed no difference in growth compared with the wild type strain (Supplementary Figure S2). Thus, these results confirm the role of Ecm33 in the TOR pathway, but not via a direct effect of cell wall damage. This study also tested the involvement of Ecm33 to respond to different types of stress. RML2U showed hypersensitivity to heat stress (from 37 to 45°C), oxidative stress (Menadione and H_2_O_2_) and osmotic stress, demonstrating a role for Ecm33 in resistance to these different stresses.

Ecm33 function appears to be linked with the secretion of Sap2 through the TOR signaling pathway (Gil-Bona et al., 2015b). This pathway is an important mediator of survival in a population of non-dividing cells, the CLS, in addition to other cellular responses implicated in aging, growth and morphogenesis (Wullschleger et al., 2006; Kaeberlein et al., 2007; Fontana et al., 2010). CR increases the life span of yeast and also of yeast with decreased TOR pathway signaling (Kaeberlein et al., 2005; Powers et al., 2006; Bonawitz et al., 2007; Wei et al., 2008). In optimum nutrient conditions, TOR kinase activity is stimulated, which activate growth processes and inactivate starvation responses. However, in starvation conditions, the activation of growth responses is prevented and starvation responses are activated by the inhibition of TOR kinase activity. Due to the hypersensitivity of RML2U to rapamycin, the role of Ecm33 in the TOR pathway was analyzed by CLS and CR assays. The mean life span of RML2U was reduced compared to that of SC5314 (**Figure 6**). These results agree with those observed in a large-scale survey in *S. cerevisiae*, in which the CLS of the *ecm33△* mutant decreased (Marek and Korona, 2013). In *C. albicans*, only three genes have been implicated in a reduced CLR to date: a protein required for respiratory growth, Growth and Oxidant Adaptation (Goa1), and two protein kinases related to the TOR pathway, Rim15 and Sch9 (Li et al., 2011; Stichternoth et al., 2011). Stichternoth et al. (2011) demonstrated that Sch9 is not involved in regulating CLS in *C. albicans*, and proposed a regulatory pathway for the long-term survival of *C. albicans* that was different from that in *S. cerevisiae*. These data suggest that the reduced survival of RML2U is more related to the cell wall damage than to the TOR pathway. According to these differences in regulation, the high-osmolarity glycerol (HOG) protein is directly involved in the sensitivity of *C. albicans* to rapamycin, whereas it is not in *S. cerevisiae*, but the link between the TOR and HOG pathways remains unclear (Li et al., 2008). It is uncertain how Tor1 activity is regulated in *C. albicans*, but a recent study published a role in hyphal elongation (Su et al., 2013). Moreover, in a large-scale study, genetic and physical interactions were observed between Ecm33 and the two components of the TOR pathway present in *S. cerevisiae*, Tor1 and Tor2 (Aronova et al., 2007; Costanzo et al., 2010). Further genetic and proteomic analysis will further clarify the relationship between Tor1 and Ecm33 in *C. albicans.*

Caffeine (1,3,7-trimethylxanthine) is an analog of purine bases that is used as a phenotypic criterion to evaluate the CWI, because several mutants that are defective in components of CWI pathway are caffeine-sensitive (Martin et al., 2000; Park et al., 2005). In addition, caffeine interferes with TOR function, inhibiting the function of the kinase and extending the life span of *S. cerevisiae* (Martin et al., 2000; Park et al., 2005; Kuranda et al., 2006; Reinke et al., 2006). For these reasons, and based on previous results, the sensitivity of RML2U to caffeine was tested. As expected, RML2U was hypersensitive to caffeine. Taken together, these phenotypes together with those previously published, such as sensitivity to zymolyase and cell wall-perturbing agents, link Ecm33 function with CWI and the structure of the β-1,3-glucan network (Martínez-Lopez et al., 2004; Gil-Bona et al., 2015b). In addition, taking into account the importance of Ecm33 for “*de novo*” cell wall biosynthesis, the results suggest that Ecm33 also has a role in cell wall reconstruction.

The RML2U mutant displays an anomalous morphology in standard growth conditions (Martínez-Lopez et al., 2004). In this study, an extensive phenotypic characterization of RML2U was performed, to try to understand the pathways that are altered by the absence of *ECM33* function. The observed morphologies suggest a role for Ecm33 in cell division and cell cycle progression. A previous study, in which the function of the molecular chaperone heat shock protein 90 (Hsp90) was compromised, resulted in a similar morphology, which was attributed to cell cycle arrest (Senn et al., 2012). The same study also showed that the F-actin staining was highly polarized, whereas in this study, RML2U showed actin patches and an unusual distribution in the cell during both growth phases. Polarization of the actin cytoskeleton is required for normal cell shape, thus, Ecm33 is required for the polarized localization of actin patches. The Sur7 transmembrane protein is also required for normal actin localization and cell wall synthesis (Alvarez et al., 2008) and was also detected in the extracellular secretome of RML2U and following fluconazole treatment (Sorgo et al., 2011; Gil-Bona et al., 2015a,b). However, the role of Sur7 is different following infection by murine macrophages, and *sur7△* cells were phagocytozed less efficiently than wild-type cells (Douglas et al., 2012); in this context, RML2U was more engulfed than SC5314 at early time points, but caused less damage to macrophages than SC5314. These data agree with those of previous study (Keppler-Ross et al., 2010), which showed that both murine and bone marrow macrophages have a high preference for yeast cells that contain mannan in their cell wall to be uptaken. Martínez-Lopez et al. (2006) showed that RML2U had an abnormally electron dense outer mannoprotein layer, which might be related to the increased preference for RML2U cells by macrophages. The low damage caused to macrophages by RML2U compared to SC5314 might be due to several reasons, which relate to the phenotypes observed in the mutant, such as the sensitivity to oxidative stress, the delay in forming filaments, or temperature, among others. Apoptosis induced in *C. albicans* by macrophages was measured by two different apoptotic markers: ROS accumulation and caspase-like activity, because the combined evaluation of apoptotic methods is required, to be able to conclude that a cell is undergoing apoptosis (Carmona-Gutierrez et al., 2010). A previous study proposed a model based on combined proteome and transcriptome data obtained from SC5314 cells ingested by RAW 264.7 macrophages, which indicated that some of the changes observed at the genomic and proteomic levels might be associated with apoptosis (Fernández-Arenas et al., 2007). In fact, the study of different apoptotic markers in SC5314 cells ingested by macrophages demonstrated that approximately 30% of cells showed apoptotic death after 16 h of interaction with murine macrophages. These SC5314 data were repeated in the present study (Cabezón et al., under revision and Supplementary Figure S3). In contrast with the published data, 12% of cells contained activated caspase in RML2U cells at the final two time-points studied (6 and 8 h), and ROS accumulation was observed in 16% of cells, 8 h following interaction. Apoptosis in yeast is an essential function of the cell to maintain a balance between young and old cells, and release compounds to the medium to promote the growth of the most stable and healthy cells (Sharon et al., 2009). These data suggest that the cell death of RML2U within the macrophage is the result of different mechanisms, including apoptosis, because the percentage of cells expressing apoptotic markers is very low, and the hostile environment and the lack of available nutrients, as well as all the disadvantages caused by the lack of Ecm33 function, contribute to the inability of the mutant to cause damage to the immune system.

To conclude, the appearance of “veil growth” in static and stationary RML2U cultures is a new phenomenon. A similar structure was observed during aging of certain white wines, in which yeasts develop a film on the wine surface after alcoholic fermentations, which is known as “flor velum” yeast (Alexandre, 2013). More than 95% of the velum is composed of *S. cerevisiae* and yeasts have acquired the ability to float as an adaptative mechanism to tolerate environmental constraints. A comparative study of a *S. cerevisiae* strain that was unable to form a velum, and one that could, revealed that the main difference between both strains was the presence of a mannoprotein in the cell wall of the flor forming yeast (Alexandre et al., 2000). However, other processes observed in RML2U and not in SC5314 might be responsible for, or participate in, velum formation, such as the flocculation process, aging or aberrant morphology (**Figures 4** and **5**; Martínez-Lopez et al., 2004). In an attempt to connect the previous results, if the TOR pathway was affected because Ecm33 potentially functions upstream to the pathway, nutrient starvation due to growth on cornmeal should enhance autophagy. In fact, cornmeal medium is used to stimulate chlamydospore formation in *C. albicans*, an adaptation to survive unfavorable conditions. The function of this process is similar to autophagy, which is the adaptative response to stress and promotes cellular survival, but in some cases, promotes cell death (Kroemer et al., 2009). Possibly, RML2U cells enter the survival stage and show phenotypes similar to both autophagy and chlamydospore formation: round cells similar to chlamydospores and vacuolization and translucent cytoplasm similar to during phagocytosis. Furthermore, the RML2U cells observed by TEM are similar to those observed in a GPI-anchor synthesis mutant of *Aspergillus fumigatus*, which showed morphological evidence of autophagy (Yan et al., 2013). Massive vacuolization and translucent cytoplasm were observed by TEM in this mutant after growth for 24 and 36 h, similar to RMLU2 cells grown in cornmeal medium. Although, the nitrogen source and addition of amino acids did not rescue the wild type phenotype of RML2U cells, this morphology was even more extreme in SC5314.

## Conclusion

The data presented in this study implicate Ecm33 in cell wall biogenesis, morphogenesis, tolerance to stress-induced agents, host–pathogen interactions and survival. The exact role of this protein is still unknown, but previous published data, together with those here, suggest a role in two different pathways: the CWI and TOR pathways. In addition, the “veil growth” observed in this mutant represents a form of growth that has never been described for *C. albicans*, and is probably related to a role to adapt and survive in extreme environmental conditions.

## Author Contributions

AG-B: design of the work, acquisition, analysis, and interpretation of data for the work and draft the work.

JR-C: design of the work, acquisition, analysis, and interpretation of data for the work and draft the work.

CP-G: design of the work, acquisition, analysis, and interpretation of data for the work.

RM-L: design of the work, acquisition, analysis, and interpretation of data for the work.

LM: design of the work, interpretation of data for the work, revising it critically for important intellectual content and final approval of the version to be published. Agreement to be accountable for all aspects of the work in ensuring that questions related to the accuracy or integrity of any part of the work are appropriately investigated and resolved.

CG: revising it critically for important intellectual content and final approval of the version to be published. Agreement to be accountable for all aspects of the work in ensuring that questions related to the accuracy or integrity of any part of the work are appropriately investigated and resolved.

## Conflict of Interest Statement

The authors declare that the research was conducted in the absence of any commercial or financial relationships that could be construed as a potential conflict of interest.
