# Supplementary material for: The Cell Wall Protein Ecm33 of Candida albicans is Involved in Chronological Life Span, Morphogenesis, Cell Wall Regeneration, Stress Tolerance, and Host–Cell Interaction
Source: Front Microbiol. 2016 Feb 2;7:64. doi: 10.3389/fmicb.2016.00064 (PMC4735633; doi:10.3389/fmicb.2016.00064)
Supplement: Supplementary file 1 [file Data_Sheet_1.DOCX]

Supplementary Material

The cell wall protein Ecm33 of *Candida albicans* is involved in chronological life span, morphogenesis, cell wall regeneration, multi-stress tolerance and host-cell interaction

**Ana Gil-Bona, Jose Antonio Reales-Calderon, Claudia Marcela-Parra, Raquel Martinez-Lopez, Lucia Monteoliva* and Concha Gil**

*** Correspondence:** Lucia Monteoliva: [luciamon@ucm.es](mailto:luciamon@ucm.es)

# Supplementary Figures and Tables

**1.1 Supplementary Figures: 1.** RML2U cells show abnormal morphologies in standard growth conditions. **2.** Deletion of different genes related to cell wall not increase susceptibility to rapamycin. **3.** Measurement of the apoptotic status of SC5314 and RML2U *C. albicans* strains after the interaction with murine macrophages.

**1.2 Supplementary Tables: 1.** Number of RML2U aberrant cells. **2.** Survival rate after CLS.

## Supplementary Figures

**
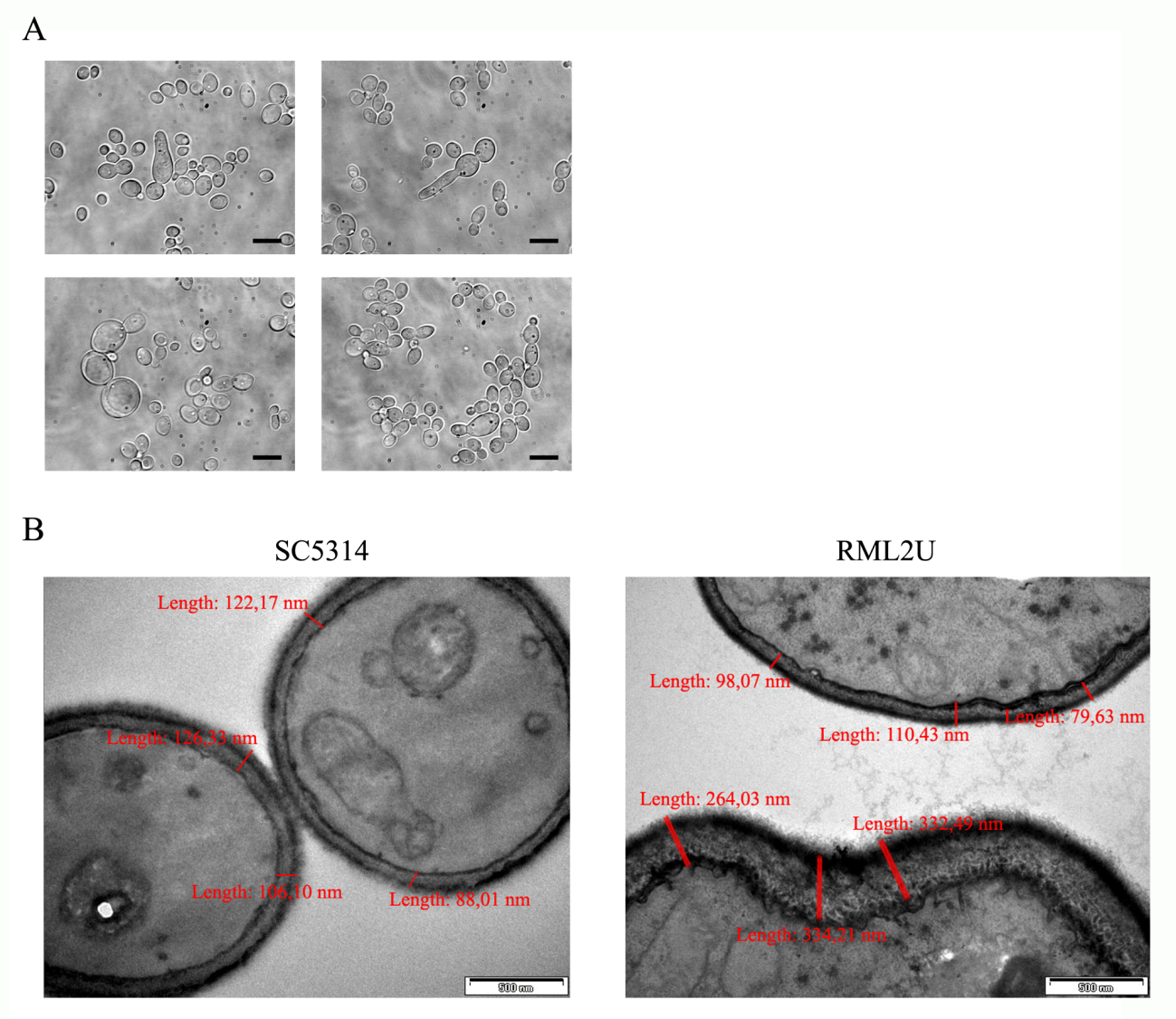
**

**Supplementary Figure 1. RML2U cells show abnormal morphologies in standard growth conditions.** (**A**) Different morphologies of RML2U cells growing in YPD medium at 30 ˚C in exponential phase. (**B**) Representative TEM images of SC5314 and RML2U cells obtained from YPD cultures at 30 °C during the exponential phase. Red lines indicate the thickness of the cell wall. Scale bars: 500 nm


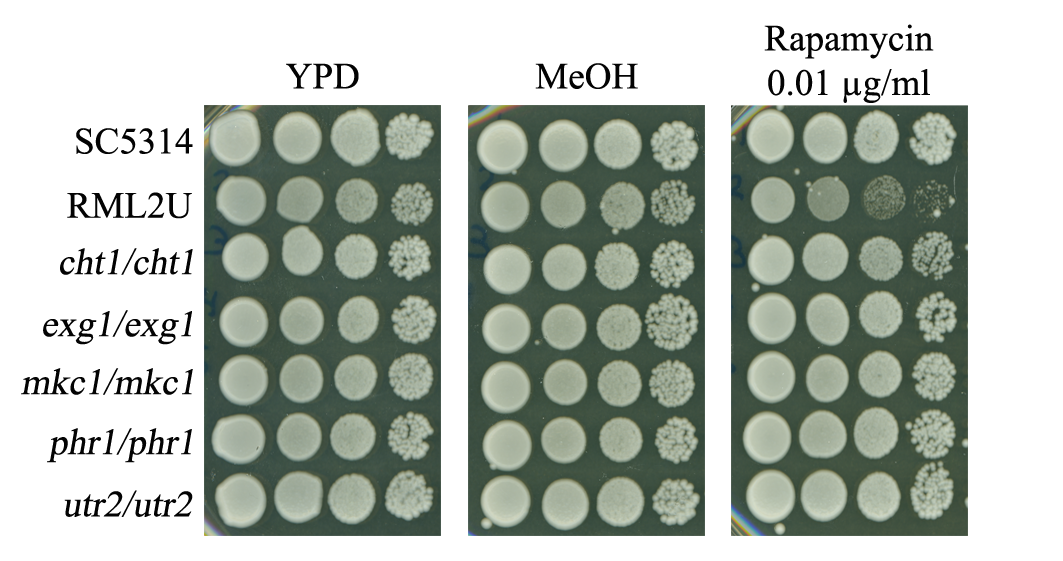


**Supplementary Figure 2. Deletion of different genes related to cell wall not increase susceptibility to rapamycin.** 10-fold serial dilutions of strains SC5314 (wild type), RML2U (*ecm33/ecm33*), *cht1/cht1*, *exg1/exg1*, *mkc1/mkc1*, *phr1/phr1* and *utr2/utr2* of *C. albicans* were spotted onto YPD with 0.01 μg/mL rapamycin or with MeOH (as control). Plates were incubated at 30 °C for 24 h. Data are representative of at least three independent experiments with identical results.


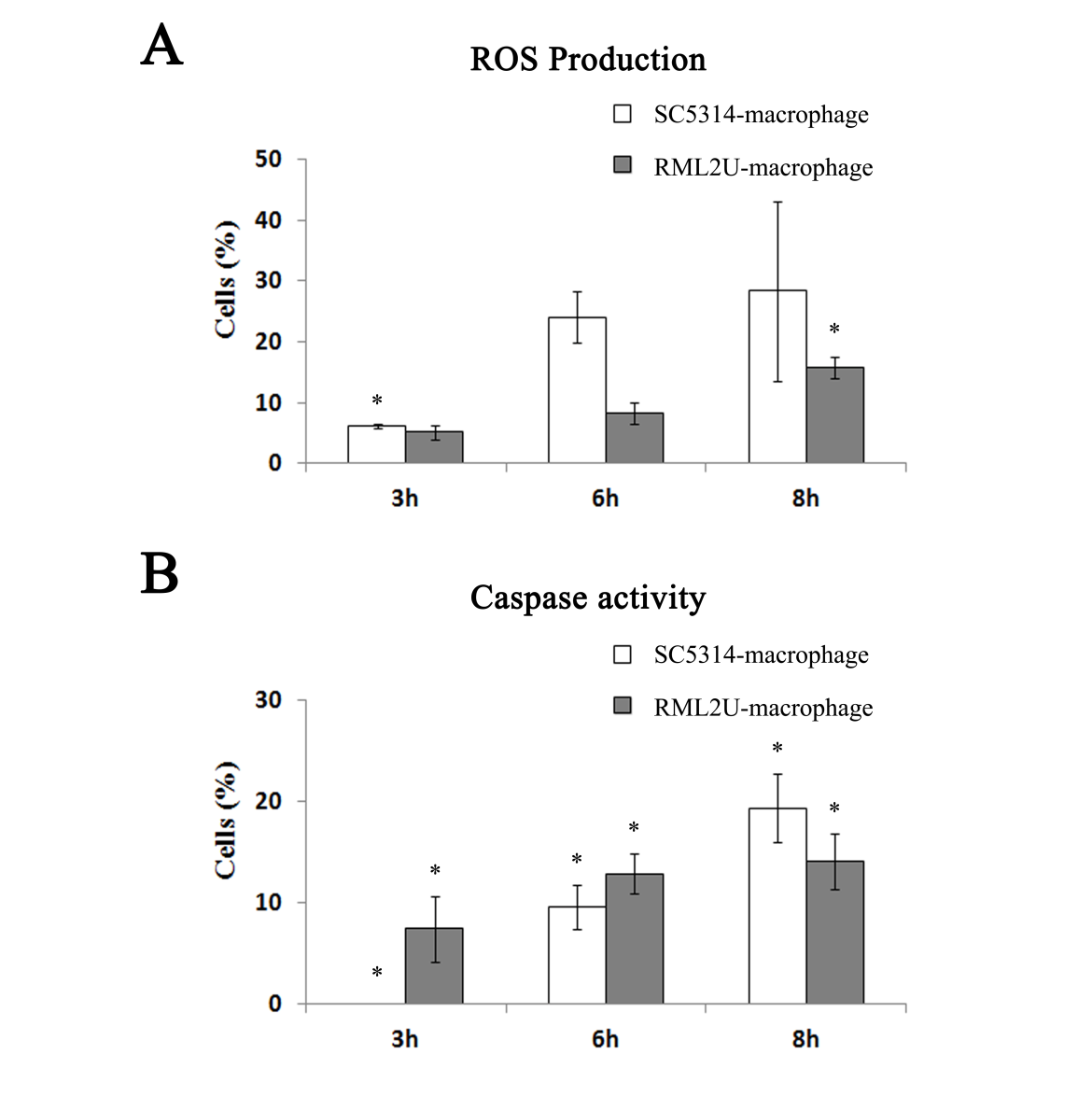


**Supplementary Figure 3. Measurement of the apoptotic status of SC5314 and RML2U *C. albicans* strains after the interaction with murine macrophages.** (**A**) Percentage of *C. albicans* cells that contain ROS after 3 h, 6 h and 8 h macrophage interaction. (**B**) Percentage of *C. albicans* cells with active caspasa after 3 h, 6 h and 8 h macrophage interaction. (*, p < 0.05).

## Supplementary Tables

**Supplementary Table 1. Number of RML2U aberrant cells.** Different morphologies resulted from compromised *ECM33* function were observed in cells growing in YPD medium at 30 °C by nomarski microscopy. The number of aberrant cells was counted and the percentage was obtained.

|  |  | Aberrant morphology | No DNA detected | More than 1 nucleus | Total aberrant cells (total cells observed) |
| --- | --- | --- | --- | --- | --- |
| Exponential Phase | **Number of observed cells** | 94 | 20 | 4 | 118 (922) |
|  | **Percentage** | 10% | 2% | 0.43% | 12.43% |
| Stationary Phase | **Number of observed cells** | 106 | 23 | 3 | 132 (509) |
|  | **Percentage** | 21% | 4.51% | 0.58% | 25.82% |

**Supplementary Table 2. Survival rate after CLS.** The number of colonies at day 3 was considered to be the initial survival (100%). Cells of each strain were collected every 3 days and plated on YPD agar for 48 h to determine % survival. The percentages showed in the table correspond to the end of the study (day 30).

|  | **Survival rate after treatment** | |
| --- | --- | --- |
|  | **SC5314** | **RML2U** |
| **Chronological Life Span (CLS)** | 0.7% | 0.08% |
| **0.5 % Glucose SD** | 24.7% | 2.4% |
| **CLS under Extreme Calorie Restriction – Water** | 32.26% | 28.83% |
